# (*E*)-2-[(6-Ethoxy­benzothia­zol-2-yl)imino­meth­yl]-6-methoxy­phenol

**DOI:** 10.1107/S1600536809009337

**Published:** 2009-03-25

**Authors:** Ling-Qian Kong

**Affiliations:** a Dongchang College, Liaocheng University, 250059 Liaocheng, Shandong, People’s Republic of China

## Abstract

In the title mol­ecule, C_17_H_16_N_2_O_3_S, the benzothia­zole fragment and the benzene ring form a dihedral angle of 13.8 (4)°, and an intramolecular O—H⋯N hydrogen bond occurs. In the crystal structure, pairs of weak inter­molecular O—H⋯S and C—H⋯(O,O) hydrogen bonds link mol­ecules into centrosymmetric dimers. These dimers are related by translation along the *a* axis and form stacks *via* π–π inter­actions, with a short inter­molecular distance of 3.766 (5) Å between the centroids of the benzene and thia­zole rings.

## Related literature

For a related crystal structure, see: Zhao *et al.* (2008 ▶). For details of the crystallography and coordination chemistry of Schiff base compounds, see: Garnovski *et al.* (1993 ▶).
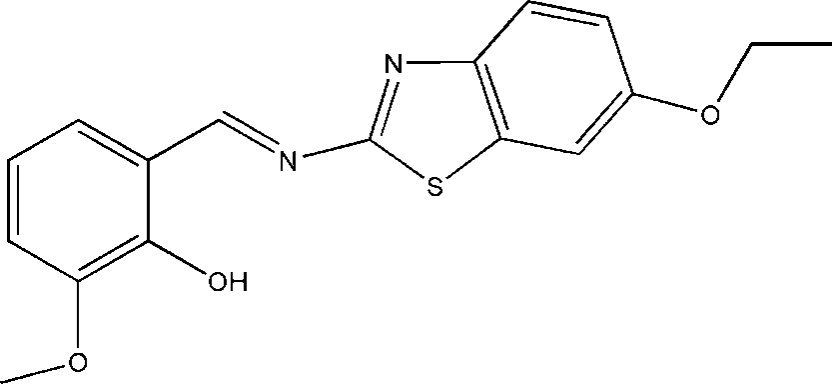


## Experimental

### 

#### Crystal data

C_17_H_16_N_2_O_3_S
*M* *_r_* = 328.38
Triclinic, 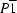

*a* = 6.0178 (14) Å
*b* = 10.941 (3) Å
*c* = 12.164 (3) Å
α = 85.479 (4)°
β = 83.693 (5)°
γ = 76.486 (3)°
*V* = 772.9 (3) Å^3^
*Z* = 2
Mo *K*α radiation
μ = 0.23 mm^−1^
*T* = 298 K
0.12 × 0.08 × 0.06 mm

#### Data collection

Bruker SMART APEX diffractometer
Absorption correction: multi-scan (*SADABS*; Sheldrick, 1996 ▶) *T*_min_ = 0.973, *T*_max_ = 0.987
4102 measured reflections
2720 independent reflections
1911 reflections with *I* > 2σ(*I*)
*R*_int_ = 0.020

#### Refinement

*R*[*F* ^2^ > 2σ(*F* ^2^)] = 0.044
*wR*(*F* ^2^) = 0.114
*S* = 1.03
2720 reflections
209 parameters
H-atom parameters constrained
Δρ_max_ = 0.18 e Å^−3^
Δρ_min_ = −0.21 e Å^−3^

### 

Data collection: *SMART* (Siemens, 1996 ▶); cell refinement: *SAINT* (Siemens, 1996 ▶); data reduction: *SAINT*; program(s) used to solve structure: *SHELXS97* (Sheldrick, 2008 ▶); program(s) used to refine structure: *SHELXL97* (Sheldrick, 2008 ▶); molecular graphics: *SHELXTL* (Sheldrick, 2008 ▶); software used to prepare material for publication: *SHELXTL*.

## Supplementary Material

Crystal structure: contains datablocks global, I. DOI: 10.1107/S1600536809009337/cv2526sup1.cif

Structure factors: contains datablocks I. DOI: 10.1107/S1600536809009337/cv2526Isup2.hkl

Additional supplementary materials:  crystallographic information; 3D view; checkCIF report

## Figures and Tables

**Table 1:** Hydrogen-bond geometry (Å, °)

*D*—H⋯*A*	*D*—H	H⋯*A*	*D*⋯*A*	*D*—H⋯*A*
O1—H1⋯N1	0.82	1.88	2.606 (3)	147
O1—H1⋯S1^i^	0.82	2.92	3.1746 (18)	100
C12—H12⋯O1^i^	0.93	2.59	3.328 (3)	136
C12—H12⋯O2^i^	0.93	2.60	3.491 (3)	160

Symmetry code: (i) 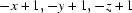.

**Fig. 1.**
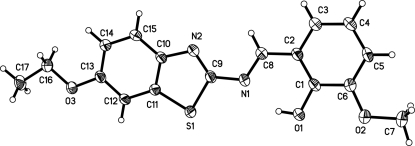
The molecular structure of (I) showing 30% probability displacement ellipsoids and the atom-numbering scheme.

## supplementary crystallographic information

### Comment

Recently, a number of Schiff base compounds have been investigated in terms of their crystallography and coordination chemistry (Garnovski *et al.*, 1993). In order to continue our studies on Schiff bases, we now report the synthesis and crystal structure of the title compound, (I).

In (I) (Fig. 1), all the geometric parameters are in a good agreement with those found in (*E*)-2-methoxy-6-[(5-methylisoxazol-3-yl)-iminomethyl] phenol (Zhao *et al.*, 2008). The benzene and the benzothiazole rings make a dihedral angle of 13.8 (4)° showing that the Schiff base ligand adopts a non-planar conformation in the case. Moreover, weak intermolecular O—H···S and C—H···O hydrogen bonds (Table 1) link the molecules into centrosymmetric dimers. These dimers related by translation along axis *a* form stacks *via*π–π interactions proved by short intermolecular distance of 3.766 (5) Å between the centroids of benzene and thiazole rings.

### Experimental

The title compound was synthesized by the reaction of 2-hydroxy-3-methoxybenzaldehyde (0.152 g, 1 mmol) and 6-ethoxybenzothiazol-2-amine (0.194 g, 1 mmol) in ethanol solution and stirred under reflux conditions (353 K) for 5 h. When cooled to room temperature the solution was filtered and after a week yellow crystals suitable for X-ray diffraction study were obtained. Yield, 0.283 g, 86%. m.p. 342–344 K.

### Refinement

The H atoms were included in the riding-model approximation with C—H = 0.93 Å, C—H = 0.96 Å and O—H = 0.82 Å, and with *U*_iso_(H) = 1.2*U*_eq_(C-aromatic) and *U*_iso_(H) = 1.5*U*_eq_(C-methyl, methylene and O).

### Crystal data

**Table:**

C_17_H_16_N_2_O_3_S	*Z* = 2
*M**_r_* = 328.38	*F*(000) = 344
Triclinic, *P*1	*D*_x_ = 1.411 Mg m^−^^3^
Hall symbol: -P 1	Mo *K*α radiation, λ = 0.71073 Å
*a* = 6.0178 (14) Å	Cell parameters from 982 reflections
*b* = 10.941 (3) Å	θ = 2.6–22.3°
*c* = 12.164 (3) Å	µ = 0.23 mm^−^^1^
α = 85.479 (4)°	*T* = 298 K
β = 83.693 (5)°	Block, yellow
γ = 76.486 (3)°	0.12 × 0.08 × 0.06 mm
*V* = 772.9 (3) Å^3^	

### Data collection

**Table:**

Bruker SMART APEX diffractometer	2720 independent reflections
Radiation source: fine-focus sealed tube	1911 reflections with *I* > 2σ(*I*)
graphite	*R*_int_ = 0.020
φ and ω scans	θ_max_ = 25.1°, θ_min_ = 1.7°
Absorption correction: multi-scan (*SADABS*; Sheldrick, 1996)	*h* = −7→7
*T*_min_ = 0.973, *T*_max_ = 0.987	*k* = −12→12
4102 measured reflections	*l* = −14→9

### Refinement

**Table:**

Refinement on *F*^2^	Primary atom site location: structure-invariant direct methods
Least-squares matrix: full	Secondary atom site location: difference Fourier map
*R*[*F*^2^ > 2σ(*F*^2^)] = 0.044	Hydrogen site location: inferred from neighbouring sites
*wR*(*F*^2^) = 0.114	H-atom parameters constrained
*S* = 1.03	*w* = 1/[σ^2^(*F*_o_^2^) + (0.0548*P*)^2^ + 0.0301*P*] where *P* = (*F*_o_^2^ + 2*F*_c_^2^)/3
2720 reflections	(Δ/σ)_max_ < 0.001
209 parameters	Δρ_max_ = 0.18 e Å^−^^3^
0 restraints	Δρ_min_ = −0.21 e Å^−^^3^

### Special details

**Table:**

Geometry. All e.s.d.'s (except the e.s.d. in the dihedral angle between two l.s. planes) are estimated using the full covariance matrix. The cell e.s.d.'s are taken into account individually in the estimation of e.s.d.'s in distances, angles and torsion angles; correlations between e.s.d.'s in cell parameters are only used when they are defined by crystal symmetry. An approximate (isotropic) treatment of cell e.s.d.'s is used for estimating e.s.d.'s involving l.s. planes.
Refinement. Refinement of *F*^2^ against ALL reflections. The weighted *R*-factor *wR* and goodness of fit *S* are based on *F*^2^, conventional *R*-factors *R* are based on *F*, with *F* set to zero for negative *F*^2^. The threshold expression of *F*^2^ > σ(*F*^2^) is used only for calculating *R*-factors(gt) *etc*. and is not relevant to the choice of reflections for refinement. *R*-factors based on *F*^2^ are statistically about twice as large as those based on *F*, and *R*- factors based on ALL data will be even larger.

### Fractional atomic coordinates and isotropic or equivalent isotropic displacement parameters (Å^2^)

**Table:**

	*x*	*y*	*z*	*U*_iso_*/*U*_eq_	
S1	0.46082 (11)	0.40652 (6)	0.37670 (5)	0.0489 (2)	
O1	0.8898 (3)	0.68081 (19)	0.43648 (14)	0.0622 (5)	
H1	0.8120	0.6363	0.4157	0.093*	
O2	1.1451 (3)	0.83142 (17)	0.47463 (15)	0.0609 (5)	
O3	−0.0448 (3)	0.15300 (16)	0.22235 (13)	0.0528 (5)	
N1	0.7555 (3)	0.54862 (17)	0.29849 (16)	0.0439 (5)	
N2	0.5822 (3)	0.45416 (18)	0.16878 (16)	0.0447 (5)	
C1	1.0271 (4)	0.7137 (2)	0.3503 (2)	0.0437 (6)	
C2	1.0358 (4)	0.6701 (2)	0.2444 (2)	0.0413 (6)	
C3	1.1832 (4)	0.7085 (2)	0.1588 (2)	0.0498 (7)	
H3	1.1915	0.6787	0.0886	0.060*	
C4	1.3155 (4)	0.7898 (2)	0.1772 (2)	0.0539 (7)	
H4	1.4111	0.8161	0.1194	0.065*	
C5	1.3068 (4)	0.8329 (2)	0.2824 (2)	0.0518 (7)	
H5	1.3978	0.8877	0.2945	0.062*	
C6	1.1657 (4)	0.7957 (2)	0.3686 (2)	0.0458 (6)	
C7	1.2911 (5)	0.9082 (3)	0.5008 (2)	0.0687 (9)	
H7A	1.4484	0.8668	0.4822	0.103*	
H7B	1.2668	0.9221	0.5786	0.103*	
H7C	1.2566	0.9876	0.4593	0.103*	
C8	0.8943 (4)	0.5867 (2)	0.2223 (2)	0.0436 (6)	
H8	0.9036	0.5596	0.1511	0.052*	
C9	0.6154 (4)	0.4739 (2)	0.2685 (2)	0.0413 (6)	
C10	0.4252 (4)	0.3792 (2)	0.17276 (19)	0.0406 (6)	
C11	0.3392 (4)	0.3421 (2)	0.27927 (19)	0.0397 (6)	
C12	0.1811 (4)	0.2670 (2)	0.2947 (2)	0.0418 (6)	
H12	0.1246	0.2435	0.3655	0.050*	
C13	0.1098 (4)	0.2280 (2)	0.2018 (2)	0.0423 (6)	
C14	0.1905 (4)	0.2659 (2)	0.0958 (2)	0.0473 (6)	
H14	0.1394	0.2394	0.0343	0.057*	
C15	0.3456 (4)	0.3422 (2)	0.0812 (2)	0.0477 (6)	
H15	0.3963	0.3686	0.0103	0.057*	
C16	−0.1092 (4)	0.1015 (2)	0.1306 (2)	0.0520 (7)	
H16A	0.0253	0.0516	0.0905	0.062*	
H16B	−0.1827	0.1684	0.0805	0.062*	
C17	−0.2723 (4)	0.0206 (2)	0.1744 (2)	0.0595 (8)	
H17A	−0.1959	−0.0472	0.2216	0.089*	
H17B	−0.3230	−0.0133	0.1137	0.089*	
H17C	−0.4023	0.0703	0.2160	0.089*	

### Atomic displacement parameters (Å^2^)

**Table:**

	*U*^11^	*U*^22^	*U*^33^	*U*^12^	*U*^13^	*U*^23^
S1	0.0572 (4)	0.0570 (4)	0.0411 (4)	−0.0323 (3)	−0.0019 (3)	0.0001 (3)
O1	0.0725 (13)	0.0819 (14)	0.0470 (11)	−0.0529 (11)	0.0118 (9)	−0.0104 (10)
O2	0.0722 (13)	0.0712 (12)	0.0521 (11)	−0.0419 (10)	−0.0002 (9)	−0.0115 (10)
O3	0.0598 (11)	0.0620 (11)	0.0476 (10)	−0.0381 (9)	0.0015 (8)	−0.0062 (9)
N1	0.0431 (11)	0.0474 (12)	0.0461 (12)	−0.0220 (10)	−0.0008 (10)	−0.0009 (10)
N2	0.0443 (12)	0.0506 (12)	0.0429 (12)	−0.0211 (10)	0.0007 (9)	−0.0006 (10)
C1	0.0410 (13)	0.0452 (14)	0.0464 (15)	−0.0183 (12)	0.0033 (11)	0.0027 (12)
C2	0.0393 (13)	0.0419 (14)	0.0443 (14)	−0.0154 (11)	−0.0021 (11)	0.0033 (11)
C3	0.0490 (15)	0.0566 (16)	0.0474 (15)	−0.0225 (13)	0.0015 (12)	−0.0016 (13)
C4	0.0519 (16)	0.0616 (17)	0.0519 (16)	−0.0289 (14)	0.0097 (13)	0.0014 (14)
C5	0.0498 (15)	0.0530 (16)	0.0602 (17)	−0.0306 (13)	0.0020 (13)	−0.0012 (14)
C6	0.0473 (14)	0.0462 (14)	0.0473 (15)	−0.0183 (12)	−0.0024 (12)	−0.0025 (12)
C7	0.081 (2)	0.0717 (19)	0.0689 (19)	−0.0453 (17)	−0.0054 (16)	−0.0165 (16)
C8	0.0402 (13)	0.0486 (15)	0.0432 (14)	−0.0146 (12)	0.0006 (11)	−0.0032 (12)
C9	0.0391 (13)	0.0418 (14)	0.0453 (15)	−0.0158 (11)	−0.0010 (11)	−0.0010 (12)
C10	0.0390 (13)	0.0435 (14)	0.0418 (14)	−0.0177 (11)	0.0031 (11)	−0.0028 (11)
C11	0.0414 (13)	0.0401 (13)	0.0394 (13)	−0.0140 (11)	−0.0008 (11)	−0.0035 (11)
C12	0.0450 (14)	0.0446 (14)	0.0390 (13)	−0.0208 (12)	0.0046 (11)	−0.0012 (11)
C13	0.0403 (13)	0.0423 (14)	0.0475 (15)	−0.0181 (11)	0.0011 (11)	−0.0024 (12)
C14	0.0487 (15)	0.0580 (16)	0.0409 (14)	−0.0245 (13)	0.0004 (11)	−0.0070 (12)
C15	0.0490 (15)	0.0600 (16)	0.0381 (14)	−0.0248 (13)	0.0042 (11)	−0.0014 (12)
C16	0.0538 (16)	0.0576 (16)	0.0527 (16)	−0.0279 (13)	−0.0038 (13)	−0.0075 (13)
C17	0.0587 (17)	0.0564 (17)	0.0731 (19)	−0.0333 (14)	−0.0030 (15)	−0.0058 (15)

### Geometric parameters (Å, °)

**Table:**

S1—C11	1.732 (2)	C5—H5	0.9300
S1—C9	1.743 (2)	C7—H7A	0.9600
O1—C1	1.342 (3)	C7—H7B	0.9600
O1—H1	0.8200	C7—H7C	0.9600
O2—C6	1.361 (3)	C8—H8	0.9300
O2—C7	1.425 (3)	C10—C15	1.383 (3)
O3—C13	1.370 (3)	C10—C11	1.407 (3)
O3—C16	1.417 (3)	C11—C12	1.385 (3)
N1—C8	1.289 (3)	C12—C13	1.382 (3)
N1—C9	1.396 (3)	C12—H12	0.9300
N2—C9	1.293 (3)	C13—C14	1.394 (3)
N2—C10	1.383 (3)	C14—C15	1.380 (3)
C1—C2	1.399 (3)	C14—H14	0.9300
C1—C6	1.405 (3)	C15—H15	0.9300
C2—C3	1.397 (3)	C16—C17	1.500 (3)
C2—C8	1.443 (3)	C16—H16A	0.9700
C3—C4	1.370 (3)	C16—H16B	0.9700
C3—H3	0.9300	C17—H17A	0.9600
C4—C5	1.390 (4)	C17—H17B	0.9600
C4—H4	0.9300	C17—H17C	0.9600
C5—C6	1.373 (3)		
			
C11—S1—C9	88.69 (11)	N2—C9—N1	126.4 (2)
C1—O1—H1	109.5	N2—C9—S1	117.12 (17)
C6—O2—C7	117.7 (2)	N1—C9—S1	116.38 (18)
C13—O3—C16	117.82 (18)	C15—C10—N2	124.9 (2)
C8—N1—C9	118.2 (2)	C15—C10—C11	119.1 (2)
C9—N2—C10	109.4 (2)	N2—C10—C11	115.9 (2)
O1—C1—C2	122.7 (2)	C12—C11—C10	121.7 (2)
O1—C1—C6	117.7 (2)	C12—C11—S1	129.48 (19)
C2—C1—C6	119.6 (2)	C10—C11—S1	108.82 (16)
C3—C2—C1	119.4 (2)	C13—C12—C11	118.0 (2)
C3—C2—C8	119.6 (2)	C13—C12—H12	121.0
C1—C2—C8	121.1 (2)	C11—C12—H12	121.0
C4—C3—C2	120.6 (2)	O3—C13—C12	115.4 (2)
C4—C3—H3	119.7	O3—C13—C14	123.7 (2)
C2—C3—H3	119.7	C12—C13—C14	120.9 (2)
C3—C4—C5	120.0 (2)	C15—C14—C13	120.6 (2)
C3—C4—H4	120.0	C15—C14—H14	119.7
C5—C4—H4	120.0	C13—C14—H14	119.7
C6—C5—C4	120.8 (2)	C14—C15—C10	119.6 (2)
C6—C5—H5	119.6	C14—C15—H15	120.2
C4—C5—H5	119.6	C10—C15—H15	120.2
O2—C6—C5	125.7 (2)	O3—C16—C17	107.7 (2)
O2—C6—C1	114.7 (2)	O3—C16—H16A	110.2
C5—C6—C1	119.7 (2)	C17—C16—H16A	110.2
O2—C7—H7A	109.5	O3—C16—H16B	110.2
O2—C7—H7B	109.5	C17—C16—H16B	110.2
H7A—C7—H7B	109.5	H16A—C16—H16B	108.5
O2—C7—H7C	109.5	C16—C17—H17A	109.5
H7A—C7—H7C	109.5	C16—C17—H17B	109.5
H7B—C7—H7C	109.5	H17A—C17—H17B	109.5
N1—C8—C2	121.9 (2)	C16—C17—H17C	109.5
N1—C8—H8	119.0	H17A—C17—H17C	109.5
C2—C8—H8	119.0	H17B—C17—H17C	109.5

### Hydrogen-bond geometry (Å, °)

**Table:**

*D*—H···*A*	*D*—H	H···*A*	*D*···*A*	*D*—H···*A*
O1—H1···N1	0.82	1.88	2.606 (3)	147
O1—H1···S1^i^	0.82	2.92	3.1746 (18)	100
C12—H12···O1^i^	0.93	2.59	3.328 (3)	136
C12—H12···O2^i^	0.93	2.60	3.491 (3)	160

Symmetry codes: (i) −*x*+1, −*y*+1, −*z*+1.
